# PKM2 regulates neural invasion of and predicts poor prognosis for human hilar cholangiocarcinoma

**DOI:** 10.1186/s12943-015-0462-6

**Published:** 2015-11-14

**Authors:** Guanzhen Yu, Wenlong Yu, Guangzhi Jin, Dongyun Xu, Ying Chen, Tian Xia, Allan Yu, Wenzheng Fang, Xiaoli Zhang, Zhaosheng Li, Keping Xie

**Affiliations:** Department of Oncology, East Hospital, Tongji University School of Medicine, Shanghai, 200120 People’s Republic of China; Department of Surgery, Eastern Hepatobiliary Hospital, Shanghai, People’s Republic of China; Department of Pathology, Eastern Hepatobiliary Hospital, Shanghai, People’s Republic of China; Department of Pathology, Changhai Hospital, Shanghai, People’s Republic of China; Department of Gastroenterology, Changhai Hospital, Shanghai, 200433 People’s Republic of China; Department of Gastroenterology, Hepatology and Nutrition, Unit 1466, The University of Texas MD Anderson Cancer Center, 1515 Holcombe Boulevard, Houston, TX 77030 USA; Department of Pathology, Chinese People‘s Liberation Army, No 411 Hospital, Shanghai, People’s Republic of China

**Keywords:** Prognosis, Metabolism, Biomarkers, PKM2, hilar cholangiocarcinoma

## Abstract

**Background:**

The therapeutic and prognostic value of the glycolytic enzymes hexokinase, phosphofructokinase, and pyruvate kinase (PK) has been implicated in a variety of cancers, while their roles in treatment of and prognosis for hilar cholangiocarcinoma (HC) remain unclear. In this study, we determined the expression of PKM2 in and its impact on biology and clinical outcome of human HC.

**Methods:**

The regulation and function of PKM2 in HC pathogenesis was evaluated using human tissues, molecular and cell biology, and animal models, and its prognostic significance was determined according to its impact on patient survival.

**Results:**

We found that expression of hexokinase 1 and the M2 splice isoform of PK (PKM2) was upregulated in HC tissues and that this expression correlated with tumor recurrence and outcome. PKM2 expression was increased in HC cases with chronic cholangitis as demonstrated by isobaric tags for relative and absolute quantification. High PKM2 expression was highly correlated with high syndecan 2 (SDC2) expression and neural invasion. PKM2 downregulation led to a decrease in SDC2 expression. Treatment with metformin markedly suppressed PKM2 and SDC2 expression at both the transcriptional and posttranscriptional levels and inhibited HC cell proliferation and tumor growth.

**Conclusions:**

PKM2 regulates neural invasion of HC cells at least in part via regulation of SDC2. Inhibition of PKM2 and SDC2 expression contributes to the therapeutic effect of metformin on HC. Therefore, PKM2 is an independent prognostic factor and potential therapeutic target for human HC.

**Electronic supplementary material:**

The online version of this article (doi:10.1186/s12943-015-0462-6) contains supplementary material, which is available to authorized users.

## Background

Hilar cholangiocarcinoma (HC) is a complex, aggressive disease with an extremely poor prognosis. R0 resection offers the only chance for long-term survival of HC. Even after resection, 5-year survival rates for HC range from 10 to 40 %, and the recurrence rates range from 50 to 70 % [1]. The most accepted prognostic factors for HC include lymph node metastasis, disease stage, negative margins, and/or neural invasion (NI) [1]. Tumor markers, specifically, carcinoembryonic antigen and CA19-9, help with diagnosis, treatment, and monitoring the progression of HC [2]. Additionally, the presence of tumor markers is associated with disease progression and overall survival (OS) [2–4]. However, physicians have successfully applied few of these biomarkers to clinical practice. Therefore, identifying novel biomarkers is of great importance to monitoring progression of, predicting outcome of, and developing effective therapeutic targets for HC.

Cancer cells can switch oxidative phosphorylation to aerobic glycolysis, known as the Warburg effect, to maintain the growth and survival of cancer cells [5]. Investigators have well established that accelerated aerobic glycolysis is associated with activation of oncogenes or inhibition of tumor suppressors in certain cells [6]. Three rate-limiting enzymes active in glycolysis—hexokinase (HK), pyruvate kinase (PK), and phosphofructokinase (PFK)—are widely activated in cancer cells [7]. HK2, which is highly expressed in cancer cells, is thought to be required for tumor initiation and maintenance, and HK2 deletion is therapeutic in mouse models of cancer [8]. Few studies have demonstrated that HK1 is associated with therapeutic effectiveness against cancer [9, 10]. Unlike other PK isoforms, PKM2 contributes to tumorigenesis [11]. Elevated PKM2 expression correlates with poor outcomes of cancer [12]. In addition, expression of 6-phosphofructo-2-kinase/fructose-2,6-biphosphatase 4 (PFKFB4), an isoform of PFK2, is elevated in tumors and an important regulator of cancer cell survival [13]. Additionally, key regulators of aerobic glycolysis are implicated to play roles in predicting outcome and monitoring drug resistance of HC. Therefore, targeting cancer metabolism seems to be a promising therapeutic approach [14].

Continuous progress in research of the cancer metabolic program has increased our understanding of cancer biology and helped identify novel therapeutic targets and/or predictive biomarkers. However, little is known about the status of the key enzymes HK, PK, and PFK in HC pathogenesis and treatment. However, researchers rarely have explored the therapeutic and prognostic value of glycolytic molecules, including HK, PFKB, and PKM2, regarding HC. Therefore, in the present study, we sought to determine the prognostic value of these three glycolytic enzymes in HC cases and investigated the possible mechanisms underlying PKM2’s promotion of HC growth and NI.

## Results

### Increased HK1 and PKM2 expression in HC samples

**Fig. 1:**
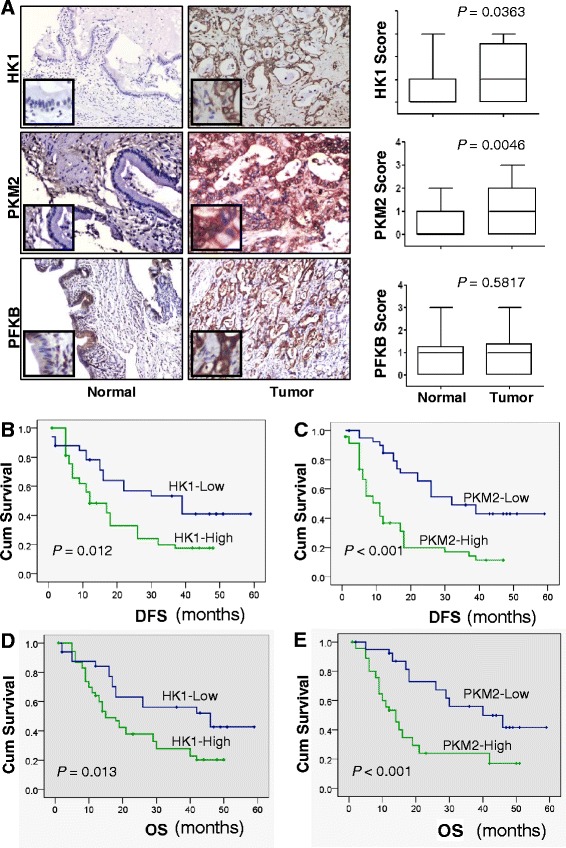
HK1, PKM2, and PFKB expression and its association with survival durations in patients with HC. **a**, HK1, PKM2, and PFKB expression in normal bile duct tissue (left panels), tumors (middle panels), and quantitative representations staining (right panels). Original magnification, ×400. **b**, patients with high HK1 expression had a shorter median time to recurrence than did those with low HK1 expression (12 months versus 39 months). Cum, cumulative. **c**, patients with high PKM2-expressing tumors had a significantly shorter median time to recurrence than did patients with low PKM2-expressing tumors (11 months versus 32 months). **d**, the median OS duration was significantly worse in patients with high expression of HK1 (15 months) than in those with low expression of HK1 (46 months). **e**, the median OS duration was significantly worse in patients with high expression of PKM2 (14 months) than in those with low expression of PKM2 (40 months)

Immunohistochemical analysis found that HK1 and PKM2, but not PFKB expression, was higher in tumor than in normal tissue samples (Fig. 1). Expression of HK1 and PKM2 protein was markedly higher in tumor than in noncancerous tissue samples (Fig. 1a). The rates of high HK1 and PKM2 expression were 63 % (55/88) and 53 % (47/88), respectively. High PFKB was expressed in 56 % (49/88) cases, but its expression pattern did not correlate with normal versus tumor tissue alone. These results were further confirmed by Western blotting which revealed a higher expression of HK1 and PKM2, but not PFKB expression, in tumor than in normal tissue samples (Additional file 1: Fig. S1)*.*

### Correlation of High HK1 and PKM2 expression with clinicopathological characteristics in HC patients

The association of high HK1, PKM2, and PFKB expression with clinicopathological factors for HC is shown in Additional file 2: Table S1. PFKB expression was associated with T1 and T2 tumors (*P* = 0.014). High HK1 expression was significantly associated with lymph node metastasis (*P* < 0.001) and stage III and IV disease (*P* = 0.006). High PKM2 expression was associated with NI (*P* = 0.001), lymph node metastasis (*P* = 0.001), and poor or lack of tumor differentiation (*P* = 0.001) (Additional file 2: Table S1). These data revealed that HK1 and PKM2 expression is significantly correlated with lymph node metastasis of HC but that PFKB expression is not. Additionally, PKM2 expression correlated significantly with NI of HC.

### Correlation of high HK1 and PKM2 expression with recurrence and survival in HC patients

The median disease-free survival (DFS) duration in the HC patients was 14 months, whereas the median OS duration was 16 months. Patients with high HK1-expressing tumors had significantly shorter median DFS durations than did patients without them (12 months versus 39 months; *P* = 0.012) (Fig. 1b). Similarly, patients with high PKM2-expressing tumors had significantly shorter DFS durations than did patients without them (11 months versus 32 months; *P* < 0.001) (Fig. 1c). High PFKB expression was not associated with DFS duration (Additional file 1: Fig. S2A). Other factors associated with HC recurrence in our univariate analysis were tumor invasion (*P* < 0.001), surgery type (*P* < 0.001), lymph node metastasis (*P* = 0.002), tissue differentiation (*P* = 0.017), and TNM category (*P* = 0.019).

As for OS, HC patients with high HK1-expressing tumors had a median duration of 15 months, whereas patients without them had a median duration of 46 months (*P* = 0.013) (Fig. 1d). Similarly, patients with high PKM2-expressing tumors had a shorter median survival duration than did those without them (14 months versus 40 months; *P* < 0.001) (Fig. 1e). High PFKB expression did not correlate with OS duration (Additional file 1: Fig. S2B). The other valuable factors in univariate analysis of OS were tumor invasion (*P* < 0.001), lymph node metastasis (*P* = 0.002), surgery type (*P* < 0.001), tissue differentiation (*P* = 0.017), and TNM category (*P* = 0.016).

### Expression of PKM2, but not HK1, as an independent prognostic factor in HC patients

Multivariate analysis demonstrated that T category, lymph node metastasis, surgery type, and high PKM2 expression were independent predictors of HC recurrence (Additional file 2: Table S2) and independent prognostic factors (Additional file 2: Table S3). Furthermore, subgroup analysis of PKM2 expression according to TNM category revealed that patients with high PKM2-expressing tumors had shorter DFS durations than did those without such tumors in each category (Additional file 1: Fig. S3A and S3B). Similarly, HC patients with high PKM2-expressing tumors had a shorter median survival duration than did patients without them in every TNM category (Additional file 1: Fig. S3C and S3D). Therefore, PKM2 appears to be a more sensitive biomarker than HK1 in monitoring HC recurrence and predicting HC outcome.

### Upregulation of PKM2 expression in patients with inflammation-related HC

Given evidence that biliary tract stone disease is an established HC risk factor, we analyzed protein expression changes in tumor samples obtained from patients with HC and biliary tract stone disease and compared them with those in inflammatory biliary tract tissue samples using an isobaric tags for relative and absolute quantification assay. We identified changes in the expression of 60 proteins using MS analysis (data not shown). PKM2 expression was higher in the tumor samples than in the noncancerous in the inflammatory tissue samples. These data further supported a potential role for PKM2 in the initiation of HC (Additional file 1: Fig. S4).

### Direct correlation of PKM2 protein expression with HC progression

**Fig. 2:**
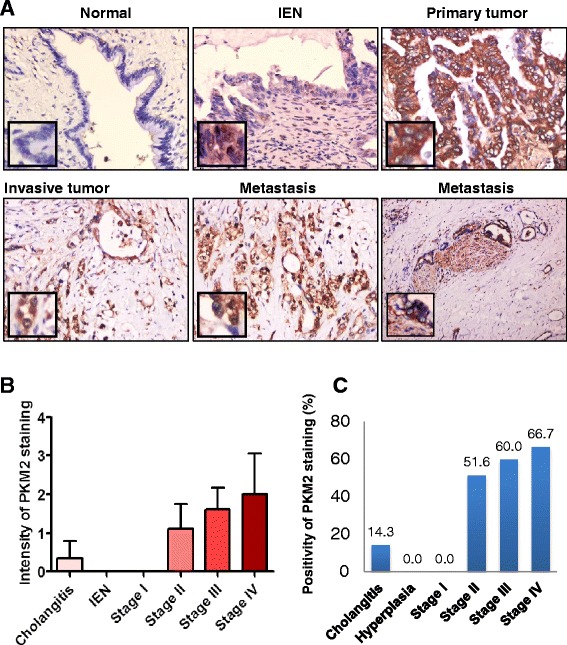
PKM2 protein expression profiles in tissue samples obtained from patients with HC at different stages. **a**, expression of PKM2 in cholangitis, biliary intraepithelial neoplastic lesion (IEN), primary tumor, invasive tumor, and metastatic tumor samples. **b**, the mean ± SD intensity of PKM2 staining in the samples increased with the progression of HC: 0.36 ± 0.48 in cholangitis, 0 in IEN, 0 in stage I, 1.11 ± 1.11 in stage II, 1.61 ± 1.26 in stage III, and 2.00 ± 1.25 in stage IV samples. **c**, the PKM2-positivity rates in cholangitis, hyperplasia, and stage I, II, III, and IV samples were 14 % (4/28), 0 % (0/3), 0 % (0/6), 52 % (16/31), 60 % (27/45), and 67 % (4/6), respectively

We then systemically analyzed the PKM2 expression locations and profiles in HC samples. PKM2 expression was predominantly located in the cytoplasm of HC cells (Fig. 2a). However, we observed nuclear staining for PKM2 in the invasion margins of primary tumors, metastatic lesions, and poorly differentiated and undifferentiated HC cells (Fig. 2a and Additional file 1: Fig. S5). The mean ± SD intensity of PKM2 staining increased with the progression of HC: 0.36 ± 0.48 for cholangitis, 0 for hyperplasia and stage I disease, 1.11 ± 1.11 for stage II disease, 1.6 ± 1.26 for stage III disease, and 2.00 ± 1.25 for stage IV disease (Fig. 2b). Similarly, the PKM2-positivity rates for cholangitis, hyperplasia, and stage I, II, III, and IV disease were 14 % (4/28), 0 % (0/2), 0 % (0/6), 52 % (16/31), 60 % (27/45), and 67 % (4/6), respectively (Fig. 2c). These data provided strong clinical evidence of a critical role for PKM2 expression in HC development.

### Association of high PKM2 expression with NI of HC

**Fig. 3:**
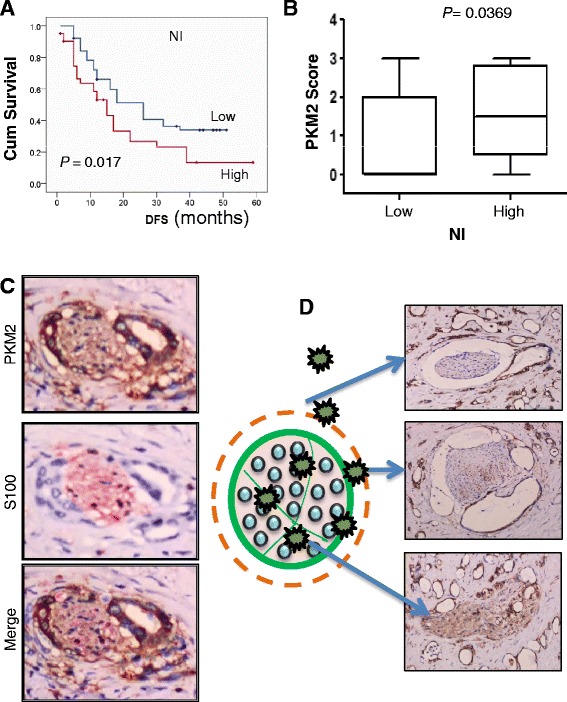
Association between PKM2 expression and PNI of HC. **a**, HC patients with PNI had a shorter median time to recurrence than did those without PNI (15 months versus 27 months). Cum, cumulative. **b**, the PKM2 staining score was higher for tumors with PNI than for tumors without PNI (*P* = 0.0369). **c**, HC cells surrounding nerve fibers (S100) exhibited strong staining for PKM2. Original magnification, ×400. D, HC cells entering the perineurium and spreading via local infiltration and metastasis all exhibited intense cytoplasmic staining for PKM2. Original magnification, ×200

Perineural invasion (PNI) is a common event in HC metastasis [15]. Our data demonstrated that the median DFS duration in HC patients without PNI was clearly longer than that in patients with PNI (26 months versus 15 months; *P* = 0.017) (Fig. 3a). Tumors with PNI had higher PKM2-positivity rates and PKM2 staining intensity than did those without PNI (Additional file 2: Table S1 and Fig. 3b). Except for those surrounding nerve fibers (Fig. 3c), all HC cells that entered the perineurium and spread via local infiltration and metastasis exhibited intense cytoplasmic staining for PKM2 (Fig. 3d). This clinical evidence demonstrated a strong association between cytoplasmic PKM2 expression and PNI of HC.

### PKM2 knockdown-attenuated NI of HC *in vivo*

**Fig. 4:**
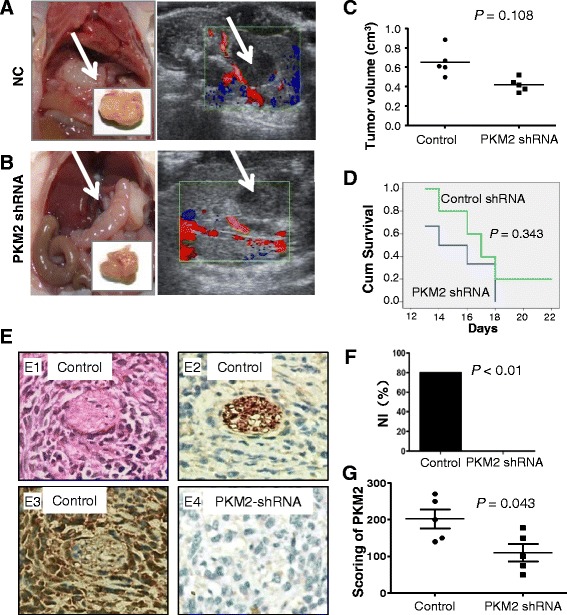
Knockdown of PKM2 expression led to inhibition of NI of HC cells in mice *in vivo*. **a** and **b**, *in situ* tumor xenograft models were used to assess the inhibitory effect of PKM2 knockdown (**a**) compared with in a negative control (NC) (**b**) as determined using anatomical (left) and ultrasonic (right) methods. **c** and **d**, tumors were weighed (**c**) and survival durations were calculated (**d**) at the end of the experiment. Cum, cumulative. **e**, histological (E1) and immunohistochemical (E2) assays demonstrated NI of tumors with high PKM2 expression (control group; E3), whereas NI of tumors with low PKM2 expression was rare (PKM2-knockdown group; E4). Original magnification, ×200. **f**, graphical representation of the rate of NI of HC in the PKM2-knockdown and control groups. **g**, graphical representation of the intensity of PKM2 staining in the PKM2-knockdown and control groups

To provide direct evidence of the critical role of PKM2 expression in NI of HC, we determined the effect of knocking down PKM2 expression with shRNA on the PNI of HC cells *in vivo*. Silencing of PKM2 expression remarkably inhibited both tumor growth and metastasis in mice as revealed by anatomical and ultrasonic assays (Fig. 4a-4c and Additional file 1: Fig. S6A and S6B). However, mice harboring high PKM2-expressing tumors did not have longer survival durations than those of mice harboring low PKM2-expressing tumors (Fig. 4d). A histopathological study demonstrated higher rates of PNI in control mice than in the PKM2-knockdown group (Fig. 4e and 4f). Immunohistochemical staining revealed a correlation between high PKM2 expression and PNI (Fig. 4e and 4g). These experimental data were consistent with our clinical observation, supporting a critical role for PKM2 expression in NI and metastasis of HC.

### PKM2-knockdown downregulated SDC2 expression

**Fig. 5:**
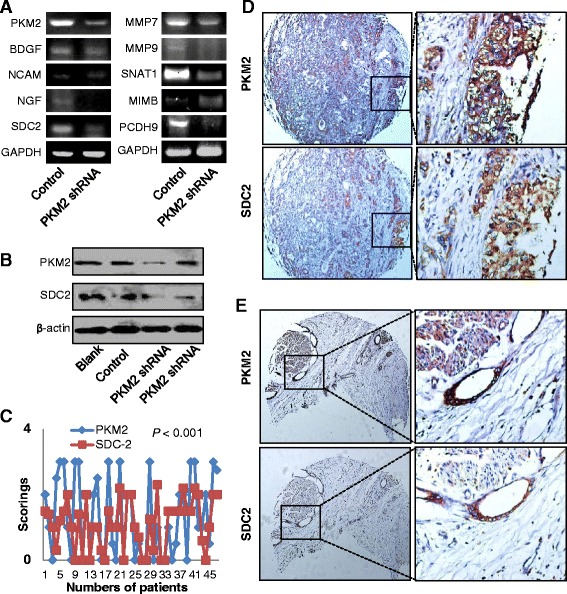
Correlation between PKM2 expression and SDC2 expression in HC cases. QBC939 cells were transiently transfected with control shRNA or PKM2 shRNA. **a** and **b**, the expression of several genes associated with NI and metastasis was measured using reverse transcription-polymerase chain reaction (**a**), and the level of PKM2 and SDC2 expression was determined using Western blotting (**b**). GAPDH, glyceraldehyde-3-phosphate dehydrogenase. **c**, graphical representation demonstrating a significant correlation between PKM2 expression and SDC2 expression in human HC tissues. **d**, co-localization of PKM2 and SDC2 in the consecutive sections of HC tissue. **e**, co-localization of PKM2 and SDC2 in the consecutive sections of HC tissue with NI. Original magnification, ×40 (left) and × 400 (right)

To determine the underlying mechanisms of PKM2’s regulation of PNI of HC, we measured the mRNA levels for molecules and metastasis-related genes possibly related to PNI. Expression of nerve growth factor (NGF), SDC2, matrix metalloproteinase (MMP) 7 and MMP9, sodium-coupled neutral amino acid transporter 1, and protocadherin 9 (PCDH9) was lower and that of metastasis suppressor 1 was higher in mice with knockdown of PKM2 expression than in the control group (Fig. 5a). Western blot analysis confirmed downregulation of SDC2 expression in QBC939 cells (Fig. 5b). As shown in Additional file 2: Table S4, we observed significant associations of SDC2 expression with NI, infiltration, and advanced stage of HC. Patients with SDC2-expressing tumors had a worse median OS duration than did those without such tumors (17 months versus 26 months; *P* = 0.025) (Additional file 1: Fig. S7).

Next, we analyzed PKM2 and SDC2 protein expression in HC cells and observed a significant correlation between the two biomarkers (*r* = 0.428; *P* < 0.001) (Fig. 5c and Additional file 2: Table S5). Furthermore, we observed co-expression of PKM2 and SDC2 in 42 % (37/88) tumors and no expression of either of them in 30 % (26/88) tumors. In the majority of the cases, PKM2 expression co-localized with SDC2 expression in the same cells from the same samples (Fig. 5d and 5e). These data provided a cross-talk between PKM2 and SDC2, which might play a role in promotion of NI of HC.

### Inhibition of HC cell growth via blockade of PKM2 and SDC2 expression by treatment with metformin

**Fig. 6:**
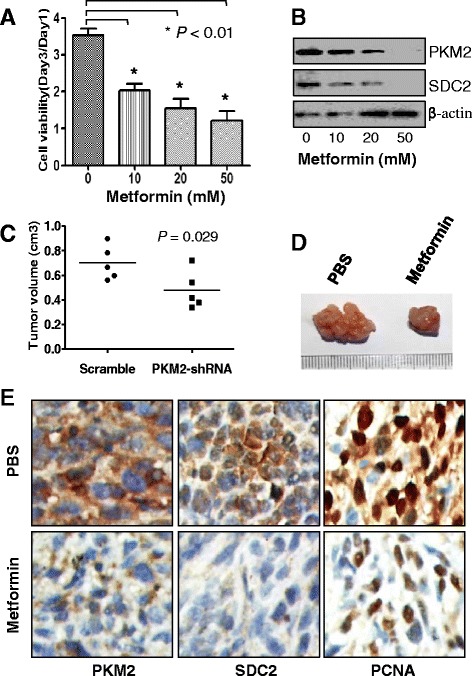
Treatment with metformin inhibited HC cell proliferation and tumor growth via suppression of PKM2 and SDC2 expression. **a**, QBC939 cells was treated with metformin at different concentrations (0, 10, 20, and 50 mM), and their viability was measured at 24 and 72 h after treatment using an MTT assay. **b**, Western blotting was used to determine the level of PKM2 and SDC2 expression after treatment with metformin. PBS, phosphate-buffered saline. **c** and **d**, *in situ* tumor xenograft models were used to assess the inhibitory effect on tumor growth of treatment with metformin (250 mg/kg) compared with that in a negative control. Tumor weights (**c**) and representative tumors from each group (**d**) were measured at the end of the experiment. **e**, PKM2, SDC2, and PCNA were detected in the xenograft tumors using immunohistochemistry

Metformin’s anticancer effects are exerted partially by blocking HK activation. Our data revealed that treatment with metformin inhibits HC cell viability in a dose-dependent manner *in vitro* (Fig. 6a and Additional file 1: Fig. S8A) and inhibits tumor growth (Fig. 6b and 6c). Also, treatment with metformin decreased both PKM2 and SDC2 expression *in vitro* and *in vivo* (Fig. 6d and 6e). These data suggested that treatment with metformin inhibited the growth of tumor cells via a novel pathway, the PKM2/SDC2 axis, supporting our hypothesis that metformin has an important role in HC treatment.

## Discussion

In this study, we systematically analyzed the expression profiles of key glycolytic enzymes in patients and mice with HC and found that expression of HK1 and PKM2 was markedly increased and correlated with tumor progression. High PKM2 expression was an independent predictor of recurrence and survival after resection of HC. Additionally, high PKM2 expression correlated directly with NI and NI-related biomarkers, especially SDC2, suggesting a novel molecular basis for the role of PKM2 in HC development and progression.

Aerobic glycolysis is essential for tumorigenesis, and the glycolytic enzymes HK, PFK, and PK are now recognized as potential therapeutic targets for cancer [14]. Researchers have also linked biological markers of glycolysis (HK2, PFKFB4, PKM2, etc.) with tumor recurrence and OS in certain cancer patients [12]. Previous studies demonstrated that expression of HK2 but not HK1 was a significant predictor of survival [16, 17]. Few studies have identified the prognostic value of PFKFB4 regarding tumor recurrence and/or survival [13]. Other studies demonstrated a significant correlation between PKM2 expression and overall prognosis for cancer [18, 19]. However, researchers rarely have explored the therapeutic and prognostic value of glycolytic molecules, including HK, PFKB, and PKM2, regarding HC. Therefore, in the present study, we investigated the HK1, PFKB, and PKM2 expression statuses in HC samples and cells and found that HK1 and PKM2 expression but not PFKB expression was higher in tumor than in normal tissue samples. Specifically, HK1 expression was associated with lymph node metastasis and advanced disease stage, whereas PKM2 expression correlated with NI, tumor invasion, lymph node metastasis, and poor or lack of tumor differentiation, suggesting that sustained aerobic glycolysis is a feature of biologically aggressive tumors. Moreover, survival analysis demonstrated that expression of HK1 and PKM2 was associated with recurrence of and prognosis for HC. However, our multivariate analysis demonstrated that expression of PKM2, but not HK1, was an independent predictor of survival of HC. Interestingly, patients with PKM2 expression had shorter times to recurrence and worse outcomes at both early and late stages of HC than did those without PKM2 expression. These findings further confirmed that aerobic glycolysis is essential for development of HC and that PKM2 is a useful biomarker for predicting HC recurrence and outcome.

A variety of risk factors for HC are well established, including chronic inflammation of biliary ducts and cirrhosis [1]. Also, a previous study identified the proteomic signatures associated with lung cancer and chronic obstructive pulmonary disease and revealed that the relevant signaling between the two diseases was related to inflammatory signaling and gluconeogenesis pathways [20]. Consistent with these findings, we observed overexpression of inflammatory signaling (ANXA4, ANXA6, etc.) (data not shown) and glycolysis (PKM2) pathways in HC and chronic inflammation of biliary ducts, suggesting that PKM2 expression contributes to tumorigenesis induced by chronic inflammatory diseases for certain cancers. Further analysis revealed that PKM2 expression was gradually upregulated as HC progressed. The predominant expression pattern was increased cytoplasmic PKM2 expression in cancer cells. However, we found nuclear staining for PKM2 at the front edges of invasion margins of primary tumor and metastatic cells. Therefore, nuclear PKM2 expression may be important to HC invasion and metastasis.

NI is highly associated with postoperative recurrence of and poor prognosis for HC [15]. Researchers have demonstrated that the molecules NGF, neural cell adhesion molecule, MMP, Ach, and transforming growth factor may predict NI of HC**.** In the present study, we found that the PKM2 expression rate in HC cases with NI was markedly higher than that in cases without NI, indicating that PKM2 may participate in the NI process. Moreover, PKM2 expression increased along with neural invasiveness of HC, and we observed cytoplasmic PKM2 expression at each step of NI, indicating a relationship between PKM2 expression and HC cells’ ability to adhere to, invade into, and survive in nervous tissue and thus metastasize via the nervous system. The critical role of PKM2 in tumorigenesis in promoting the Warburg effect makes PKM2 an attractive target for cancer therapy. However, PKM2 knockdown has not affected the growth of different types of tumors owing to the fact that slowing glycolysis generates macromolecule precursors, which support cell proliferation [14]. In our study, we observed modest impairment of survival and proliferation of HC cells after knocking down PKM2 expression. However, the rate of NI of tumors with PKM2 expression was markedly higher than that of NI of tumors without PKM2 expression. Meanwhile, PKM2 was weakly expressed in normal nervous tissue, suggesting an ectopic relationship between PKM2 expression and this tissue. These data suggest that PKM2 expression is positively correlated with NI of HC and is an indicator of NI of and prognosis for HC.

Mechanistically, in HC cells, knockdown of expression of PKM2 decreased the expression of NGF, MMP7, MMP9, and SDC2, molecules potentially related to NI. NGF facilitates cancer cell proliferation by binding to TrkA receptors in the perineurium [21]. MMP7 and MMP9 are expressed in cancer cells that invade nervous tissue [15]. SDC2 is a novel PNI-associated gene in pancreatic adenocarcinoma cases, with SDC2 silencing markedly reducing the motility and invasiveness of the cancer cells [22]. Therefore, PKM2’s mechanisms in regulating NI of HC may be associated with NGF, MMPs, and SDC2. Additionally, silencing of PKM2 leads to downregulation of expression of sodium-coupled neutral amino acid transporter 1 (a potential oncogene) [23] and upregulation of that of metastasis suppressor 1 [24], supporting a crucial role for PKM2 in tumor development and progression. However, expression of another candidate tumor suppressor, PCDH9 [25], was remarkably decreased in HC cells when PKM2 activity was blocked, suggesting an alternative mechanism in PKM2-knockdown-resistant cells.

Expression of the cell surface adhesion receptor SDC2 is increased in a variety of cancers [26, 27] and correlated with progression and poor prognosis [28]. Our data demonstrated that SDC2 expression was highly associated with progression and outcome of HC. SDC2 mediates cell adhesion and migration via several signaling pathways, such as syntenin-1, integrin α2, and RACK1 [29–31], and synthesis of SDC2 is enhanced by transforming growth factor-β and melanocortin 1 receptor [32, 33]. In the present study, we found that knockdown of PKM2 expression decreased SDC2 expression in HC cells. Clinical evidence confirmed a significant correlation between PKM2 and SDC2 expression. Furthermore, PKM2 expression co-localized with SDC2 expression in the majority of HC cells. Thirty-seven HC cases were positive for both PKM2 and SDC2, whereas 26 cases were negative for both of them, accounting for 72 % of all cases. This finding suggested that SDC2 is a novel target of PKM2 and plays an important role in regulation of HC cell migration.

Metabolic targeting has proven to be effective in preventing the development of cancer. Compounds targeting key metabolic steps in glycolysis have inhibited the proliferation, invasion, and metastasis of cancer cells [14]. Authors recently reported that treatment with metformin, an antidiabetic drug, acts on cell metabolism, inhibiting all energy-consuming processes and cell proliferation [34, 35]. Evidently, metformin directly inhibits the enzymatic function of HK1 and HK2 in breast cancer cells and markedly reduces tumor glucose consumption and growth [36]. Our present findings are consistent with those of previous studies in demonstrating the inhibitory effect of treatment with metformin on HC cell proliferation and tumor growth. Furthermore, we demonstrated that metformin decreased PKM2 and SDC2 expression in a dose-dependent manner. These results strongly suggest that inhibition of PKM2 and SDC2 expression contributes to the therapeutic potential of metformin in HC patients.

## Conclusions

PKM2 and HK1 were frequently overexpressed in HC patients, and PKM2 was an independent predictor of recurrence and OS of HC. PKM2 expression was highly correlated with NI of HC and regulated HC cell migration via SDC2. Additionally, treatment with metformin impaired the survival of HC cells by inhibiting expression of PKM2 and SDC2. These findings strongly suggest that PKM2/SDC2 signaling plays an important role in HC pathogenesis and is a promising biomarker for monitoring progression and predicting OS of HC.

## Methods

### Patient samples and cell culture

Eighty-eight patients with previously untreated HC who underwent curative surgery at Eastern Hepatobiliary Hospital or Changhai Hospital in Shanghai, People’s Republic of China, from 2004 to 2008 were enrolled in this study. The patients’ characteristics are listed in Additional file 2: Table S1. The mean age of the patients was 55 years (range, 31–79 years). Their median follow-up duration was 16 months (range, 1–59 months), with two patients dying during follow-up. Three tissue microarray blocks of tumor and matched normal bile duct tissue samples obtained from these patients were created using a manual arrayer (Beecher Instruments). Each block had two or three 1.5-mm cores of tumor tissue and one 1.5-mm core of normal bile duct tissue. The institutional review boards of both Eastern Hepatobiliary Hospital and Changhai Hospital approved the use of the patients’ samples and clinical information, and each patient or his or her guardian gave informed consent to participate in the study.

The HC cell line QBC939 was purchased from the Cell Center of the Chinese Academy of Sciences. The cells were maintained in Dulbecco’s modified Eagle’s medium with 10 % fetal bovine serum (Invitrogen) and cultured at 37 °C in 5 % CO_2_.

### Immunohistochemistry and evaluation of HC specimens

Four-micron paraffin-embedded sections of the tissue microarray blocks were prepared and processed for immunohistochemical analysis. HK1, PKM2, PFKB, S100, syndecan 2 (SDC2), and proliferating cell nuclear antigen (PCNA) protein expression in these sections was detected using appropriate antibodies against HK1 (dilution, 1:100; YT2265; ImmunoWay), PKM2 (dilution, 1:100; EPR10138[B]; Epitomics), PFKB (dilution, 1:50; YT3685; ImmunoWay), S100 (dilution, 1:100; 4C4.9; Maixin-Bio), SDC2 (dilution, 1:100; 305507; R & D Systems), and PCNA (dilution, 1:100; PC10; Maixin-Bio). A streptavidin-biotin kit (#KIT-9720; Maixin-Bio) was used to visualize antibody binding to the sections. Protein expression in the 88 cases was evaluated by two individuals (G. Yu and Y. Chen). Any discrepancy from the two individuals was resolved by a third individual. A semiquantitative scoring system was used. Specifically, the mean percentage of tumor cells positive for indicated marker(s) was calculated in five areas of a given sample at a magnification of × 400 and scored from 0 to 1 (0-100 %). The intensity of immunostaining was scored as 0 for negative, 1 for weak, 2 for moderate, and 3 for strong. Theoretically, a weighted score was generated for each case, ranging from 0 (0% of cells stained) to 300 (100 % of cells stained at 3+ intensity) [37]. The cutoff points were based on the scores: negative, 0; weak, <75; moderate, 76–150; and strong, >151 (Additional file 1: Fig. S8B). We defined the score <75 as low expression and ≥75 as high expression [38].

### Protein extraction from formalin-fixed, paraffin-embedded tissue samples; isobaric tags for relative and absolute quantification labeling; and two-dimensional liquid chromatography-electrospray ionization/multistage mass spectrometry

Proteins extracted from six inflammatory bile duct tissues and eight HC with bile duct stone disease samples were pooled separately. Protein extraction from formalin-fixed, paraffin-embedded samples; isobaric tags for relative and absolute quantification labeling two-dimensional liquid chromatography-electrospray ionization/multistage mass spectrometry (MS/MS) and a search of relevant database were carried out as described previously [39].

### Western blot analysis

Whole-cell lysates of HC cell lines, tumor samples, and matched nontumor tissue samples were prepared for Western blot analysis. Standard Western blotting [40] was performed using rabbit antibodies against human HK1 (1:1000), PKM2 (1:1000), PFKB (1:1000), and SDC2 (1:1000). Sample loading was monitored by probing the same membrane filter with an anti-β-actin antibody (sc-47778HRP; Santa Cruz Biotechnology).

### Plasmids and transfection

PKM2 short hairpin RNA (shRNA) and unspecific scrambled shRNA plasmids were gifts from Weiwei Yang (Chinese Academy of Sciences). QBC939 cells were digested, and 1 × 10^5^ of the cells were seeded in six-well plates. The cells were transfected with PKM2 or scrambled shRNA using Lipofectamine 2000 reagent (Invitrogen) and 5 ng of shRNA plasmid per well according to the reagent manufacturer’s instructions. The transfected cells were then subcultured and selected in the presence of puromycin for generation of negative control and stable PKM2-knockout cells [41].

### Cell proliferation assay

Twenty-four hours after transfection, 5000 QBC939 cells per well were seeded in 96-well plates. The parental cells were plated onto 96-well plates and treated with metformin (D6198; Sangon) at different concentrations (0, 10, 20, and 50 mM). At the indicated times, a CCK8 assay (Dojindo) was performed to assess the final results. This experiment was performed in triplicate.

### RNA isolation and reverse transcription-polymerase chain reaction

Total RNA was isolated from QBC939 HC cell lines using a total RNA isolation reagent (Advanced Biotechnologies). cDNA was generated from 1 μg of a total RNA sample using a transcription kit (Takara). An Access reverse transcription-polymerase chain reaction system (Promega) was used to measure the mRNA levels for the indicated genes and the housekeeping gene glyceraldehyde-3-phosphate dehydrogenase [42, 43].

### Animals and animal models

Orthotropic tumor xenograft models consisting of nude male Balb/c mice were used to assess both the inhibitory effect of PKM2 knockdown and effect of treatment with metformin alone. Generally, after mice were anesthetized, laparotomy was performed, and the joint of the common bile duct and portal vein were exposed. Tumor cells were injected into gelatin sponges, which were placed in the joint in the bile duct. In addition, animals were randomized into control and treatment groups (*n* = 5). Metformin-based treatment (250 mg/kg) was initiated in these mice 7 days after tumor-cell injection and was given once daily via intraperitoneal injection. The control group of mice received saline only. At 15 days after treatment, all of the mice were killed. Their primary tumors, livers, and lymph nodes were collected and processed for evaluation of gene expression. All animal experiments were approved by the Animal Ethics Committee of the Second Military Medical University [44, 45].

### Statistical analysis

Statistical analysis was performed using the SPSS statistical software (version 16.0; IBM Corporation) and Prism software (version 5.0; GraphPad Software) programs. Categorical data were analyzed using *χ*^2^ tests. Differences in protein expression between normal tissue and tumor samples were determined using the two-tailed Student *t*-test, whereas the significance of the *in vivo* data was determined using the two-tailed Mann–Whitney *U* test. Within-group correlations of variables were assessed using Pearson’s correlation coefficient. The Kaplan-Meier method was used to estimate survival rates, and the log-rank and Wilcoxon rank sum tests were performed to assess survival differences between groups. The Cox proportional hazards model for multivariate survival analysis was used to assess predictors related to survival. Two-sided *P* values less than 0.05 were considered statistically significant.

## Additional files

Additional file 1: Figure S1. PKM2 expression in HC tissues. A, Western blotting revealed the expression pattern of KK1, PFKB, and PKM2 in HC (T) and matched adjacent noncancerous normal tissue (N) (A). B, Graphical representation of the different expressions of KK1, PFKB, and PKM2 between N and T from (A) using Image J. C, Western blotting showed higher expressions of PKM2 in tumor (“T”) than normal (“N”) in another three cases of HC. **Figure S2.** PFKB expression and HC patient survival. Kaplan-Meier survival curves for patients with HC according to expression of PFKB. DFS (A) and OS (B) did not differ significantly between HC patients with low or high PFKB expression. Cum, cumulative. **Figure S3.** Subgroup survival analysis of PKM2 expression in HC patients according to TNM category. A, patients with stage I or II HC and high PKM2 expression had a shorter median time to recurrence than did those with stage I or II HC but without PKM2 overexpression. B, patients with stage III or IV HC and high PKM2 expression had a significantly shorter median time to recurrence than did patients with stage III or IV HC but with low PKM2 expression. C, the median OS duration was significantly worse in patients with stage I or II HC and PKM2 expression than in those with stage I or II HC but with low PKM2 expression. *D*, the median OS duration was significantly worse in patients with stage III or IV HC and PKM2 expression than in those with stage III or IV HC but with low PKM2 expression. Cum, cumulative. **Figure S4.** Representative MS/MS spectrum showing the peptide of PKM2 protein. A, the intensity of repots ions of precursor peptides indicating protein expression levels. N, normal bile duct tissue. B, MS/MS spectra demonstrating identified sequences of the peptide LAPITSDPTEATAVGAVEASFK leading to identification of PKM2. **Figure S5.** Localization of PKM2 expression in HC cells. A and B, cytoplasmic staining for PKM2 in well/moderately differentiated cancer cells. C and D, cytoplasmic and nuclear staining (arrow) for PKM2 in poorly differentiated cancer cells. **Figure S6.** Xenograft models of HC. A and B, tumor cells were treated with either control shRNA or PKM2 shRNA and orthotopically implanted into nude mice as described in Materials and Methods. Tumor cells with high PKM2 expression had a higher frequency of lymph node metastasis and liver lesions than did cells with low PKM2 expression (red arrows). C, tumors with downregulated PKM2 expression had lower SDC2 expression than did those with high PKM2 expression. **Figure S7.** Kaplan-Meier survival curves for patients with HC according to SDC2 expression. A, patients with high SDC2-expressing tumors had a shorter median DFS duration than did patients with low SDC2-expressing tumors. B, the median OS duration in patients with high SDC2 expression did not differ significantly from that in patients with low SDC2 expression. Cum, cumulative. **Figure S8.** Effect of metformin treatment on tumor cell growth. A, Metformin inhibited the ability of cell proliferation of RBE cells in vitro as determined by using the CCK8 assay. B, Representative photos of HC protein staining positivity and expression pattern of IHC staining were presented. (PPTX 6948 kb) Additional file 2: Table S1. Association between expression of PKM2, PFKB, and HK1 and clinical variables in HC patients. **Table S2.** Multivariate analysis of variables associated with TTP and OS in HC patients. **Table S3.** Multivariate analysis of variables associated with OS in HC patients. **Table S4.** Correlation of SDC2 expression and clinicopathological factors in HC patients. **Table S5.** Correlation between PKM2 protein overexpression and SDC2 protein overexpression in HC patients. (DOCX 40 kb)
