# Supplementary material for: PKM2 regulates neural invasion of and predicts poor prognosis for human hilar cholangiocarcinoma
Source: Mol Cancer. 2015 Nov 14;14:193. doi: 10.1186/s12943-015-0462-6 (PMC4650283; doi:10.1186/s12943-015-0462-6)
Supplement: Additional file 1: Figure S1. — PKM2 expression in HC tissues. A, Western blotting revealed the expression pattern of KK1, PFKB, and PKM2 in HC (T) and matched adjacent noncancerous normal tissue (N) (A). B, Graphical representation of the different expressions of KK1, PFKB, and PKM2 between N and T from (A) using Image J. C, Western blotting showed higher expressions of PKM2 in tumor (“T”) than normal (“N”) in another three cases of HC. Figure S2. PFKB expression and HC patient survival. Kaplan-Meier survival curves for patients with HC according to expression of PFKB. DFS (A) and OS (B) did not differ significantly between HC patients with low or high PFKB expression. Cum, cumulative. Figure S3. Subgroup survival analysis of PKM2 expression in HC patients according to TNM category. A, patients with stage I or II HC and high PKM2 expression had a shorter median time to recurrence than did those with stage I or II HC but without PKM2 overexpression. B, patients with stage III or IV HC and high PKM2 expression had a significantly shorter median time to recurrence than did patients with stage III or IV HC but with low PKM2 expression. C, the median OS duration was significantly worse in patients with stage I or II HC and PKM2 expression than in those with stage I or II HC but with low PKM2 expression. D, the median OS duration was significantly worse in patients with stage III or IV HC and PKM2 expression than in those with stage III or IV HC but with low PKM2 expression. Cum, cumulative. Figure S4. Representative MS/MS spectrum showing the peptide of PKM2 protein. A, the intensity of repots ions of precursor peptides indicating protein expression levels. N, normal bile duct tissue. B, MS/MS spectra demonstrating identified sequences of the peptide LAPITSDPTEATAVGAVEASFK leading to identification of PKM2. Figure S5. Localization of PKM2 expression in HC cells. A and B, cytoplasmic staining for PKM2 in well/moderately differentiated cancer cells. C and D, cytoplasmic and nuclear staining (arrow) for [file 12943_2015_462_MOESM1_ESM.pptx]

## Slide 1
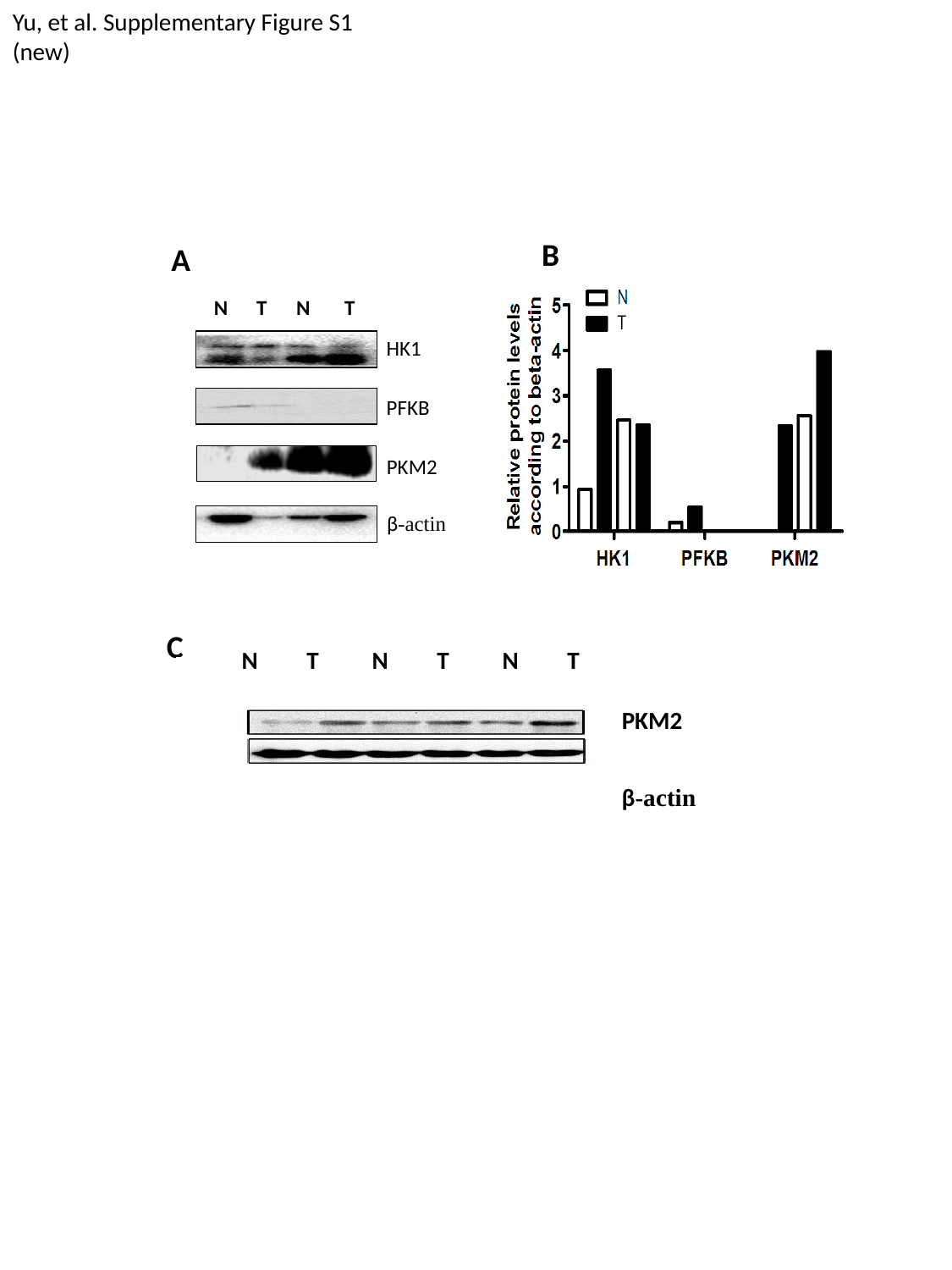

Yu, et al. Supplementary Figure S1
(new)
B
A
N
T
N
T
HK1
PFKB
PKM2
β-actin
C
N
T
N
T
N
T
PKM2
β-actin

## Slide 2
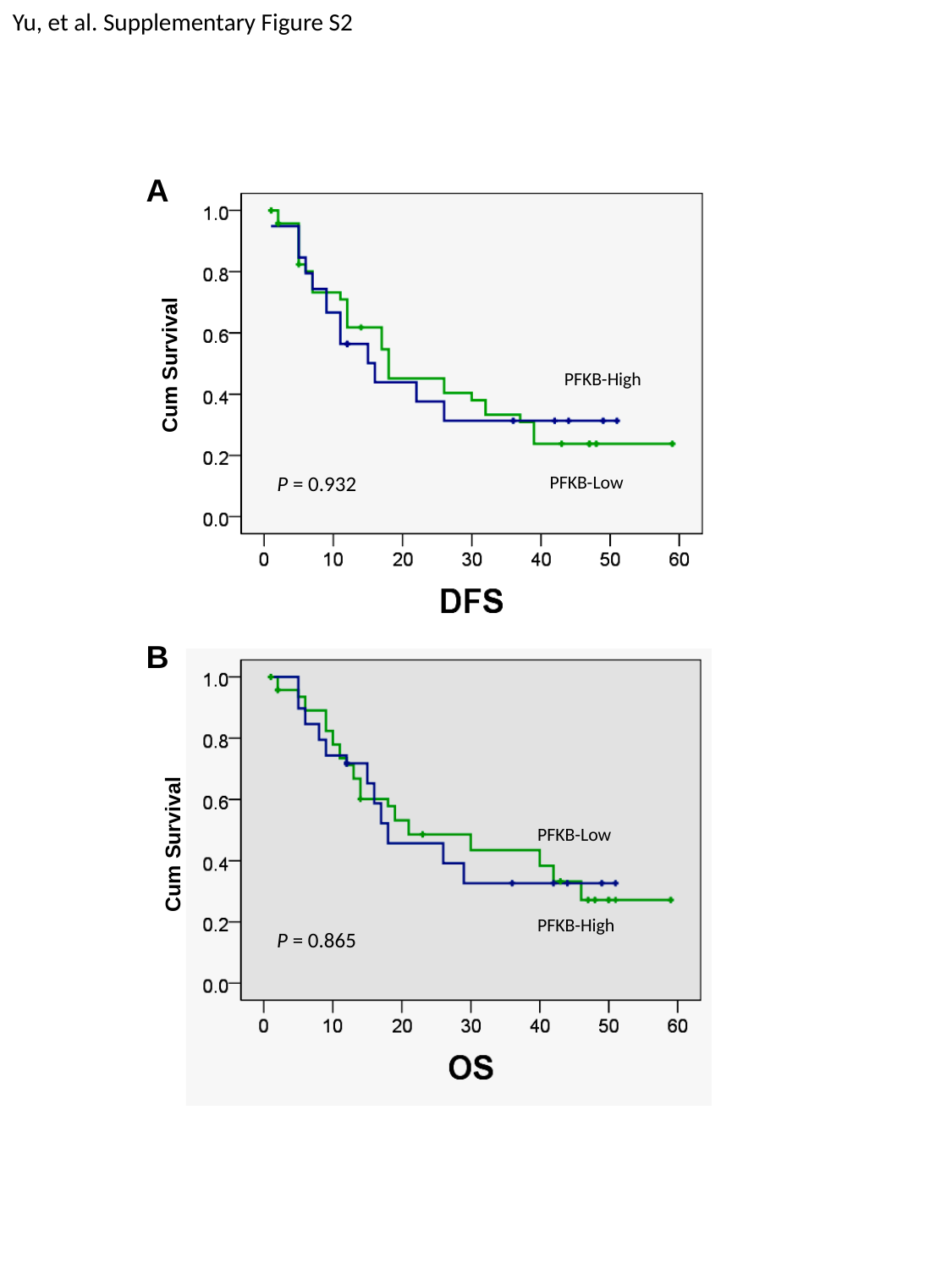

Yu, et al. Supplementary Figure S2
A
PFKB-High
P = 0.932
PFKB-Low
Cum Survival
B
PFKB-Low
PFKB-High
P = 0.865
Cum Survival

## Slide 3
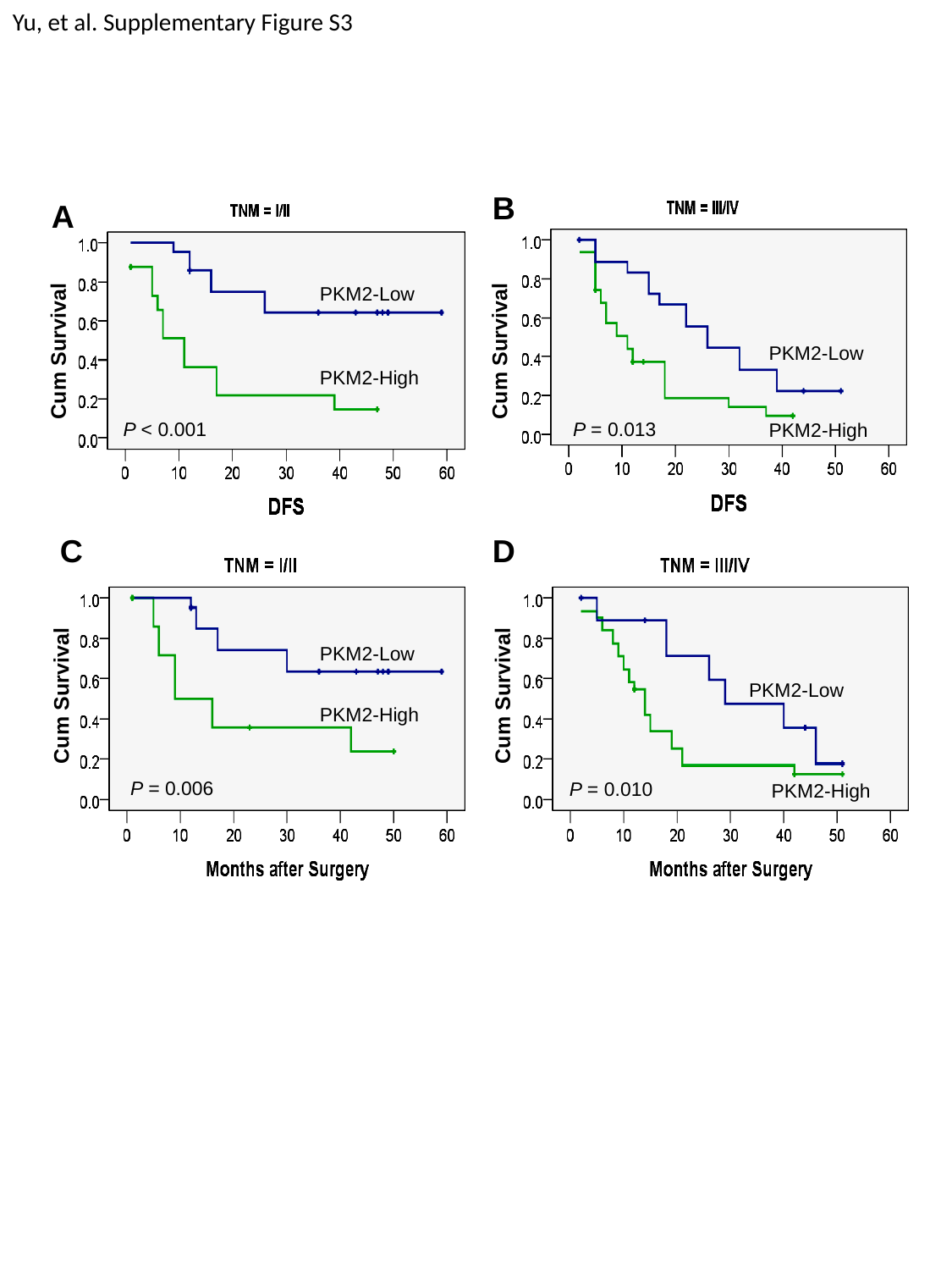

Yu, et al. Supplementary Figure S3
B
A
PKM2-Low
Cum Survival
Cum Survival
PKM2-Low
PKM2-High
P < 0.001
P = 0.013
PKM2-High
C
D
PKM2-Low
PKM2-Low
Cum Survival
Cum Survival
PKM2-High
P = 0.006
P = 0.010
PKM2-High

## Slide 4
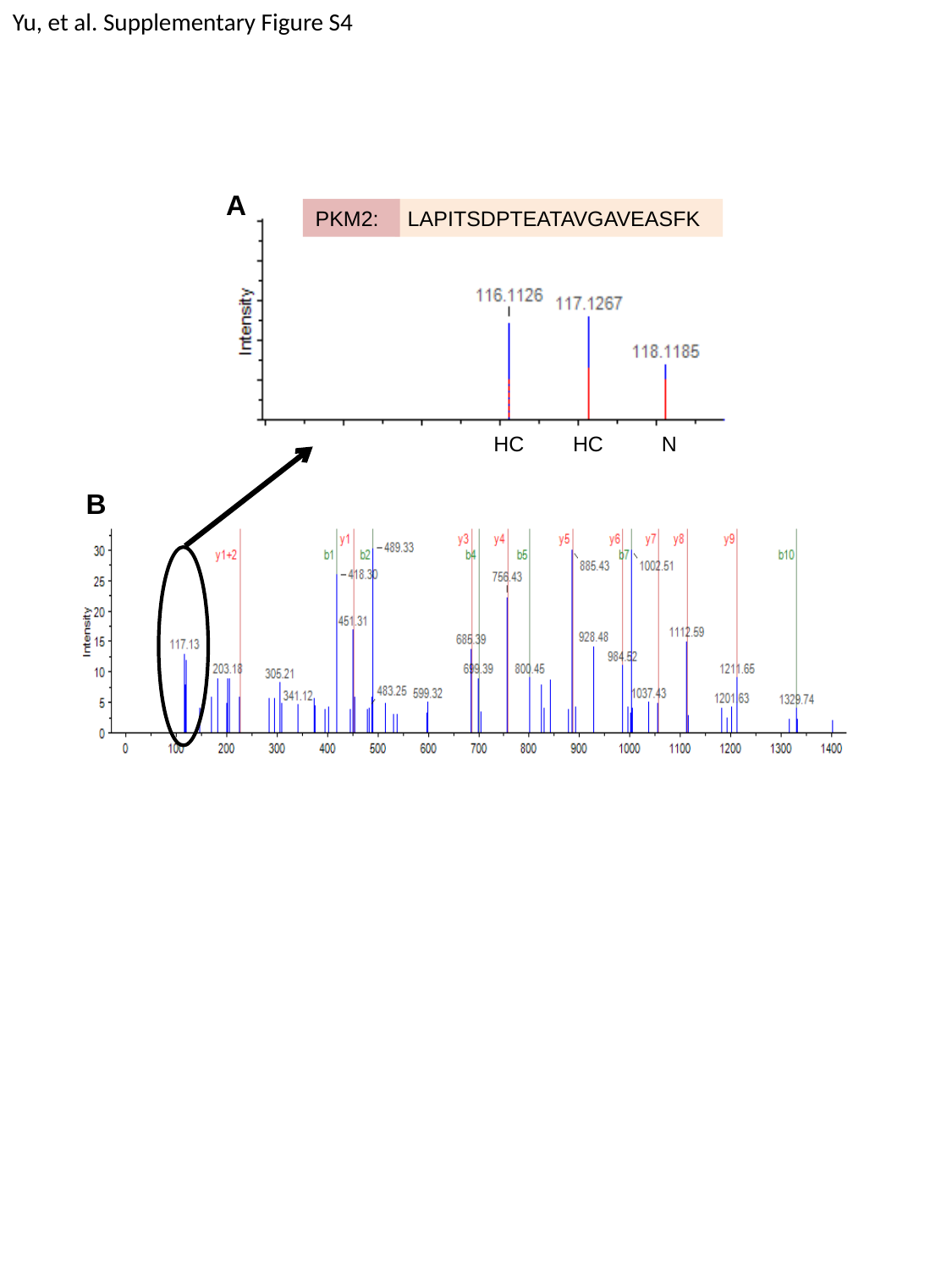

Yu, et al. Supplementary Figure S4
A
PKM2:
LAPITSDPTEATAVGAVEASFK
HC
HC
N
B

## Slide 5
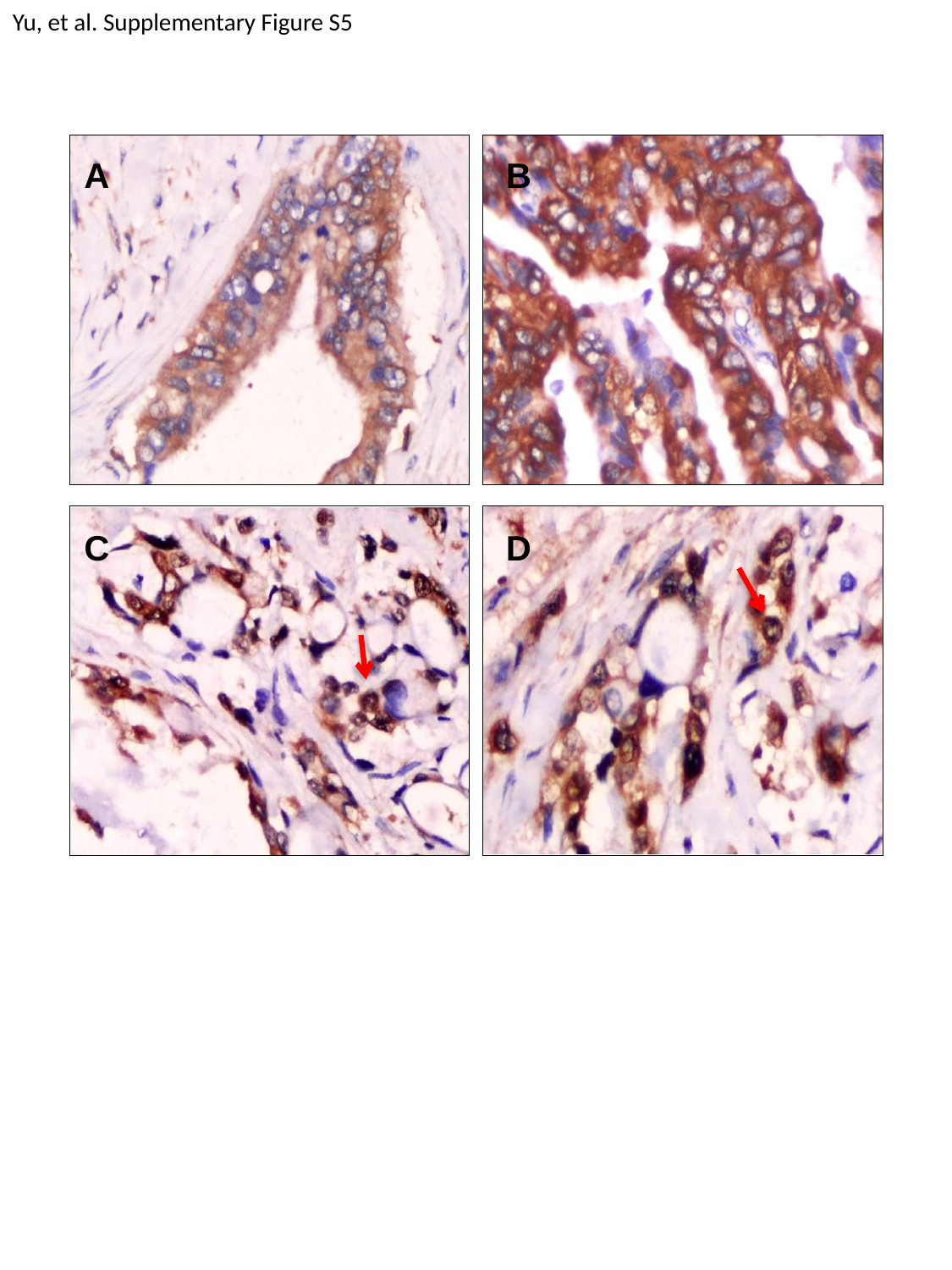

Yu, et al. Supplementary Figure S5
A
B
C
D

## Slide 6
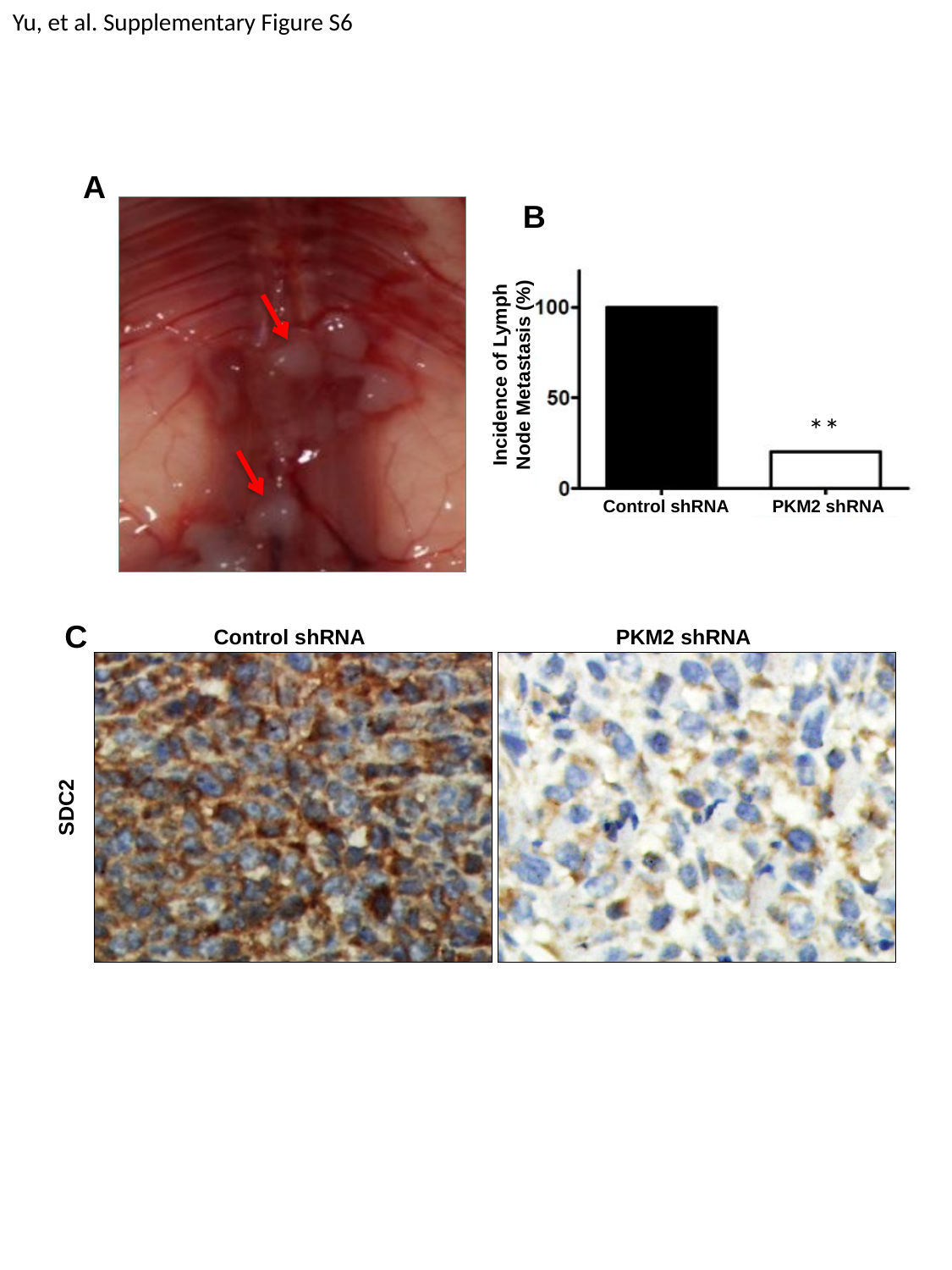

Yu, et al. Supplementary Figure S6
A
B
Incidence of Lymph
Node Metastasis (%)
**
PKM2 shRNA
Control shRNA
C
Control
PKM2 shRNA
Control shRNA
PKM2 shRNA
SDC2

## Slide 7
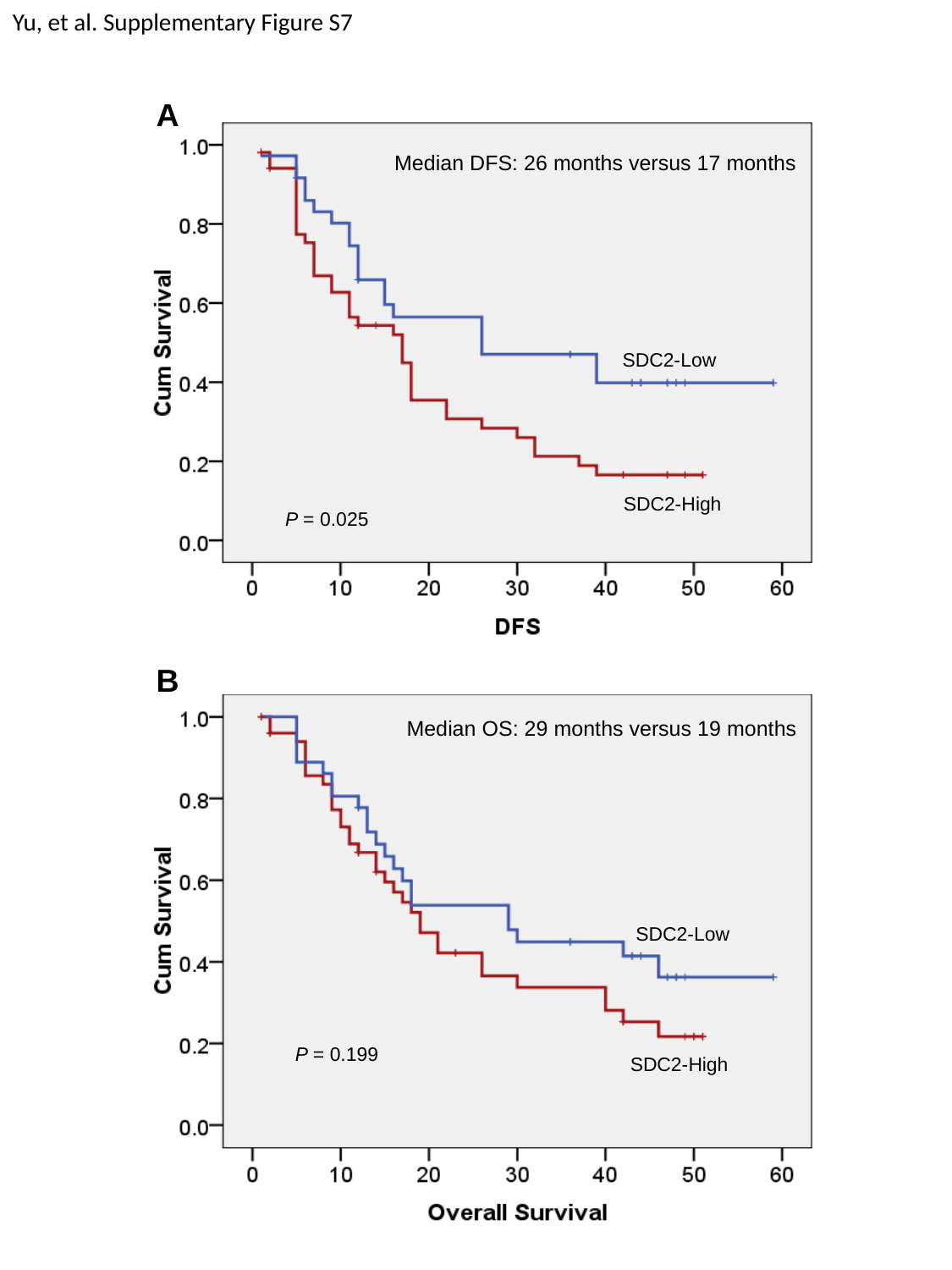

Yu, et al. Supplementary Figure S7
A
Median DFS: 26 months versus 17 months
SDC2-Low
SDC2-High
P = 0.025
B
Median OS: 29 months versus 19 months
SDC2-Low
P = 0.199
SDC2-High

## Slide 8
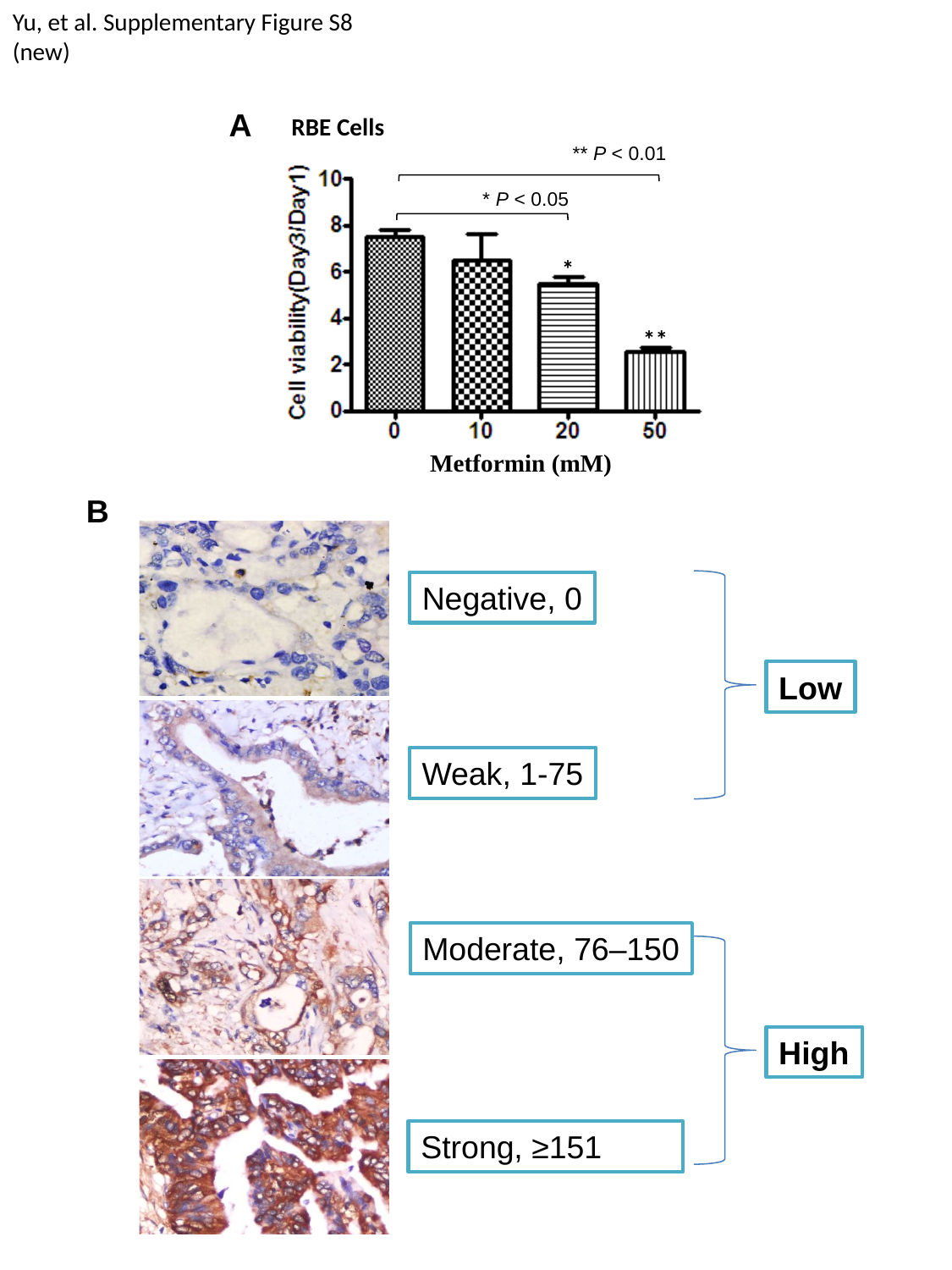

Yu, et al. Supplementary Figure S8
(new)
A
RBE Cells
** P < 0.01
* P < 0.05
*
**
Metformin (mM)
B
Negative, 0
Low
Weak, 1-75
Moderate, 76–150
High
Strong, ≥151
