# Supplementary material for: PKM2 regulates neural invasion of and predicts poor prognosis for human hilar cholangiocarcinoma
Source: Mol Cancer. 2015 Nov 14;14:193. doi: 10.1186/s12943-015-0462-6 (PMC4650283; doi:10.1186/s12943-015-0462-6)
Supplement: Additional file 2: Table S1. — Association between expression of PKM2, PFKB, and HK1 and clinical variables in HC patients. Table S2. Multivariate analysis of variables associated with TTP and OS in HC patients. Table S3. Multivariate analysis of variables associated with OS in HC patients. Table S4. Correlation of SDC2 expression and clinicopathological factors in HC patients. Table S5. Correlation between PKM2 protein overexpression and SDC2 protein overexpression in HC patients. (DOCX 40 kb) [file 12943_2015_462_MOESM2_ESM.docx]

**Supplementary Table 1.** Association between expression of PKM2, PFKB, and HK1 and clinical variables in HC patients

| **Variable** | ***n*** | **PKM2** | | ***P*** | **PFKB** | | ***P*** | **HK1** | | ***P**** |
| --- | --- | --- | --- | --- | --- | --- | --- | --- | --- | --- |
|  |  | ***n*** | **%** |  | ***n*** | **%** |  | ***n*** | **%** |  |
| Age, y |  |  |  |  |  |  |  |  |  |  |
| ≤55 | 43 | 26 | 60 | 0.195 | 26 | 60 | 0.377 | 29 | 67 | 0.349 |
| >55 | 45 | 21 | 47 |  | 23 | 51 |  | 26 | 58 |  |
| Sex |  |  |  |  |  |  |  |  |  |  |
| Male | 62 | 29 | 47 | 0.054 | 35 | 56 | 0.822 | 38 | 61 | 0.717 |
| Female | 26 | 18 | 69 |  | 14 | 54 |  | 17 | 65 |  |
| NI |  |  |  |  |  |  |  |  |  |  |
| + | 48 | 18 | 38 | 0.001 | 23 | 48 | 0.108 | 33 | 69 | 0.185 |
| - | 40 | 29 | 73 |  | 26 | 65 |  | 22 | 55 |  |
| T category |  |  |  |  |  |  |  |  |  |  |
| T1/2 | 14 | 2 | 14 | 0.001 | 12 | 86 | 0.014 | 6 | 43 | 0.098 |
| T3/4 | 74 | 45 | 61 |  | 37 | 50 |  | 49 | 66 |  |
| Nodal metastasis |  |  |  |  |  |  |  |  |  |  |
| N0 | 29 | 8 | 28 | 0.001 | 14 | 48 | 0.327 | 9 | 31 | <0.001 |
| N1 | 59 | 39 | 66 |  | 35 | 59 |  | 46 | 78 |  |
| Differentiation |  |  |  |  |  |  |  |  |  |  |
| High/moderate | 67 | 29 | 43 | 0.001 | 38 | 57 | 0.727 | 41 | 61 | 0.651 |
| Poor/undifferentiated | 21 | 18 | 86 |  | 11 | 52 |  | 14 | 67 |  |
| TNM category |  |  |  |  |  |  |  |  |  |  |
| I/II | 37 | 16 | 43 | 0.079 | 22 | 59 | 0.543 | 17 | 46 | 0.006 |
| III/IV | 51 | 31 | 61 |  | 27 | 53 |  | 38 | 75 |  |
| Total | 88 | 47 | 53 |  | 49 | 56 |  | 55 | 63 |  |

**Supplementary Table 2.** Multivariate analysis of variables associated with TTP and OS in HC patients

|  | **TTP** | | | **OS** | | |
| --- | --- | --- | --- | --- | --- | --- |
|  | ***P*** | **RR** | **95% CI** | ***P*** | **RR** | **95% CI** |
| Nerve invasion (yes versus no) | 0.280 | 0.684 | 0.344-1.362 | -0.217 | -0.638 | 0.312-1.303 |
| Tumor invasion (T1/2 versus T3/4) | 0.001 | 0.072 | 0.015-0.332 | -0.001 | -0.077 | 0.016-0.370 |
| Lymph node metastasis (yes versus no) | 0.007 | 0.371 | 0.180-0.765 | -0.038 | -0.459 | 0.220-0.957 |
| Differentiation (high/moderate versus poor/undifferentiated) | 0.714 | 1.143 | 0.560-2.332 | -0.976 | 1.011 | 0.484-2.113 |
| Surgery type (R0 versus R1/2) | 0.042 | 0.373 | 0.144-0.965 | -0.012 | 0.289 | 0.110-0.759 |
| Disease stage (I/II versus III/IV) | 0.102 | 1.847 | 0.885-3.856 | -0.098 | 1.915 | 0.886-4.138 |
| PKM2 status (High versus Low) | 0.012 | 0.402 | 0.197-0.818 | -0.043 | -0.485 | 0.241-0.976 |
| HK1 status (High versus Low) | 0.638 | 0.830 | 0.381-1.806 | -0.202 | -0.578 | 0.249-1.341 |

Abbreviations: TTP, time to progression; RR, risk ratio; CI, confidence interval; OS, overall survival.

**Supplementary Table 3.** Multivariate analysis of variables associated with OS in HC patients

| **Variable** | **B** | **SE** | **Wald** | **Sig** | **Exp (B)** | **95% CI** |
| --- | --- | --- | --- | --- | --- | --- |
| Nerve invasion (yes versus no) | -0.449 | 0.364 | 1.522 | 0.217 | 0.638 | 0.312-1.303 |
| Tumor invasion (T1/2 versus T3/4) | -2.564 | 0.800 | 10.266 | 0.001 | 0.077 | 0.016-0.370 |
| Lymph node metastasis (yes versus no) | -0.778 | 0.375 | 4.313 | 0.038 | 0.459 | 0.220-0.957 |
| Differentiation (high/moderate versus poor/undifferentiated) | 0.011 | 0.376 | 0.001 | 0.976 | 1.011 | 0.484-2.113 |
| Surgery type (R0 versus R1/2) | -1.240 | 0.492 | 6.361 | 0.012 | 0.289 | 0.110-0.759 |
| Disease stage (I/II versus III/IV) | 0.650 | 0.393 | 2.730 | 0.098 | 1.915 | 0.886-4.138 |
| PKM2 status (High versus Low) | -0.724 | 0.357 | 4.109 | 0.043 | 0.485 | 0.241-0.976 |
| HK1 status (High versus Low) | -0.548 | 0.429 | 1.627 | 0.202 | 0.578 | 0.249-1.341 |

Abbreviations: SE, standard error; CI, confidence interval.

| **Supplementary Table S4.** Correlation of SDC2 expression and clinicopathological factors in HC patients | | | | |
| --- | --- | --- | --- | --- |
| **Parameter** | ***n*** | **SDC2 expression** | | |
|  |  | ***n*** | **%** | ***P**** |
| Age, y |  |  |  |  |
| ≤55 | 43 | 26 | 60 | 0.484 |
| >55 | 45 | 26 | 58 |  |
| Sex |  |  |  |  |
| Male | 62 | 35 | 56 | 0.296 |
| Female | 26 | 17 | 65 |  |
| NI |  |  |  |  |
| + | 48 | 24 | 50 | 0.046 |
| - | 40 | 28 | 70 |  |
| T category |  |  |  |  |
| T1/2 | 14 | 2 | 14 | 0.001 |
| T3/4 | 74 | 50 | 68 |  |
| Nodal metastasis |  |  |  |  |
| N0 | 29 | 16 | 55.2 | 0.383 |
| N1 | 59 | 36 | 61.0 |  |
| Differentiation |  |  |  |  |
| High/moderate | 67 | 37 | 55 | 0.144 |
| Poor/undifferentiated | 21 | 15 | 71 |  |
| TNM category |  |  |  |  |
| I/II | 37 | 16 | 43 | 0.009 |
| III/IV | 51 | 36 | 71 |  |
| Total | 88 | 47 | 53 |  |

**Supplementary Table S5.** Correlation between PKM2 protein overexpression and SDC2 protein overexpression in HC patients

| **PKM2** | **SDC2** | | ***r*** | ***P**** |
| --- | --- | --- | --- | --- |
|  | **Low** | **High** |  |  |
| Low | 26 | 15 | 0.428 | <0.001 |
| High | 10 | 37 |  |  |
